# A Primer in Precision Nephrology: Optimizing Outcomes in Kidney Health and Disease through Data-Driven Medicine

**DOI:** 10.34067/KID.0000000000000089

**Published:** 2023-03-23

**Authors:** Pushkala Jayaraman, Andrew Crouse, Girish Nadkarni, Matthew Might

**Affiliations:** 1 The Charles Bronfman Institute for Personalized Medicine Icahn School of Medicine at Mount Sinai, New York, New York; 2 Hugh Kaul Precision Medicine Institute, University of Alabama at Birmingham, Birmingham, Alabama; 3 The Mount Sinai Clinical Intelligence Center (MSCIC), Icahn School of Medicine at Mount Sinai, New York, New York; 4 Division of Data Driven and Digital Medicine, Department of Medicine, Icahn School of Medicine at Mount Sinai, New York, New York; 5 Barbara T Murphy Division of Nephrology, Department of Medicine, Icahn School of Medicine at Mount Sinai, New York, New York; 6 Department of Medicine, University of Alabama at Birmingham, Birmingham, Alabama; 7 Department of Computer Science, University of Alabama at Birmingham, Birmingham, Alabama

**Keywords:** genetics, artificial intelligence, data science, precision medicine, precision nephrology

## Abstract

This year marks the 63rd anniversary of the International Society of Nephrology, which signaled nephrology's emergence as a modern medical discipline. In this article, we briefly trace the course of nephrology's history to show a clear arc in its evolution—of increasing resolution in nephrological data—an arc that is converging with computational capabilities to enable precision nephrology. In general, *precision medicine* refers to tailoring treatment to the individual characteristics of patients. For an operational definition, this tailoring takes the form of an optimization, in which treatments are selected to maximize a patient's expected health with respect to all available data. Because modern health data are large and high resolution, this optimization process requires computational intervention, and it must be tuned to the contours of specific medical disciplines. An advantage of this operational definition for precision medicine is that it allows us to better understand what precision medicine means in the context of a specific medical discipline. The goal of this article was to demonstrate how to instantiate this definition of precision medicine for the field of nephrology. Correspondingly, the goal of *precision nephrology* was to answer two related questions: (*1*) How do we optimize kidney health with respect to all available data? and (*2*) How do we optimize general health with respect to kidney data?

## The Path to Precision Nephrology: An Evolution of Resolution

[Authors note: Those already familiar with nephrology and its history wish to skim or skip this section. The purpose of its inclusion is to expand the audience and put the review into context for non-nephrologists.]

Before nephrology's modern form, “renal pathology” arose from Malpighi's early microscopic work in the 1600s, from Gabriel Valentin's evaluation of renal tissue with the development of thin slides,^1^ and from Friedrich Henle through the microanatomy of the kidney and the discovery of the “Loop of Henle.”^1^ “Clinical nephrology” coevolved in tandem through the work of William Bowman and Richard Bright and their study of diseased kidneys,^1^ which launched modern clinical and experimental renal pathology. *Nephrology*, the term, was coined by Jean Hamburger in the 1960s as the study of internal medicine concerning the functioning of the kidneys either in a normal healthy state or in a diseased state.^2^ The term also encompassed the preservation of kidney health, the treatment of kidney disease, and renal replacement therapy.

As nephrology has progressed, there has been a corresponding evolution in the understanding of the interaction between healthy and disease states.^3,4^ Two broad categories of disease states involving decreased kidney function capture the most common presentations of kidney-related disorders in the general population^5^: AKI covers a wide variety of pathophysiology that lead to suddenly decreased kidney function,^6^ whereas CKD refers to ongoing damage to the kidneys that slowly reduces kidney function. Either can result in end-stage renal disease requiring transplant or dialysis as well as with diminished quality of life and increased mortality risk.^7^

Although most injury to the kidney could be classified as either an acute or chronic disease state, kidney disease is more of a continuum between AKI and CKD.^8^ While increased episodes of AKI tend to increase the risk for CKD and eventual risk for end-stage kidney disease, CKD is considered a risk factor for AKI and both are now considered as risk factors for cardiovascular disease.^9,10^ Pioneering work has highlighted cardiovascular complications and also changes in bone histology with progression of CKD.^9–13^

Over the past two decades in particular, research in kidney disease epidemiology has been consolidating developmental and population health research and integrating evidence-based studies to improve kidney care.^2,13,14^ Such work has resulted in high-volume, heterogenous data,^15^ that is “Big Data” which include multiomics data, electronic health record data, pathology data. In addition, this growth toward Big Data has also prompted moves for standardization of nomenclature (as the terms “renal,” “kidney,” and “nephro” have been used interchangeably).^16,17^ Consequently, guidelines have been put in place by a special proposal by the Kidney Disease: Improving Global Outcomes (KDIGO) consortium to help reduce imprecision in patient care and to improve management of kidney health.^16^ This increase in the precision of the vocabulary is critical to formally structuring these data, which is in turn critical to leveraging all the data being produced. This will lead to an improvement in resolution of understanding of kidney health at the macro level.

## Precision Medicine and the Growth of Precision Nephrology

Since its earliest conception in a National Academies Report (2011), precision medicine has been identified with the tailoring of treatment to the individual characteristics of a patient.^18^ However, we argue that an equivalent operational definition of precision medicine should be medicine in which health is optimized with respect to data. Because the individual characteristics of the patient are included within the data being collected, this optimization process produces a natural tailoring. To be precise, identifying the true “*optimum*” would require a kind of biomedical omniscience. Thus, we must *approximate* the optimum with respect to all *available* data on a patient (as opposed to all possible data) and the total of human biomedical knowledge at that moment (as opposed to the Platonic ideal set of all biomedical knowledge). Progress in precision medicine means moving this approximation within reach of the true optimum.

On December 18, 2015, President Barack Obama launched the bipartisan *Precision Medicine Initiative*.^19^ He defined the effort as “delivering the right treatments, at the right time, every time to the right person.” The National Institutes of Health (NIH) is at the forefront of this initiative, by helping to drive participant engaged, data-driven research efforts combining biology, computation, medicine, genetics, and environmental knowledge. It is aimed at developing effective ways to improve health and treat disease at the individual or personalized level by generating the essential understanding of genotype-phenotype relationships. Within the initiative, the initial Cohort Program launched the creation of a cohort of one million participants who volunteered to add their health information and samples into a national database and biobank for large-scale biomedical and precision medicine research. Initially, the samples will provide genetic information on participants. (This Precision Medicine Initiative Cohort Program is currently known as “The All of Us Research Program.”^20,21^) *All of Us* significantly surpasses early resources, such as 1000 Genomes Project (2008–2015)^22–26^ and Genomics England's “100,000 Genomes Project^27^ in scale and scope.

With the primary emphasis in the Precision Medicine Initiative being genetic, genomic medicine and precision medicine initially became synonymous. However, the optimization process at the heart of precision medicine goes beyond genetic data, even as genetic data remain critical to the personalization aspect of precision medicine. For example, traditional clinical data, transcriptomic data, metabolomic data, and metagenomic data are all now used to complement genetic data analyses, understand disease, predict outcomes, and add individualized context to findings. This “all data” approach is critical in making precision medicine real for individual medical specialties because it enables considering our first subaim of precision nephrology: “*optimizing kidney health with respect to all available data.*”

The generation of large amounts of genomic data and newer technologies has contributed to this growing initiative in data-driven kidney medicine. In the past decade alone, the discovery of an increasing number of causal genes implicated in the development and progression of kidney disease has helped improve accuracy in renal pathology and refine insights in renal physiology.^28^ Open-source tools, such as Genome Aggregation Database (gnomAD)^29,30^ and ClinVar,^31^ enable variant interpretation through aggregation of sequenced variants at the population scale to filter through the millions of common variants within each population. gnomAD provides population-specific variant allele scores to help identify rare variants within populations. ClinVar crowd sources report of evidence for genotype-phenotype correlations while also involving a curation panel to validate some of the evidence of clinical significance of certain variants.

Integrating genetics and public health data open up the possibility of assessing the effect of identified variants in genes, such as *MYH9*,^32^ *SLC9A3R1*,^33^ or *APOL1*,^34^ as risk factors for kidney disease due to large cohort sizes and increasing diversity in population cohorts. Variants in *MYH9* are associated with 2–4 times higher risk of nondiabetic end-stage kidney disease in African populations as compared with European populations. Nephrolithiasis (NL) is highly prevalent in 10% of the individuals worldwide with poor diet and lifestyle factors playing a role in worsening symptoms^35^ and includes 30 known risk genes, including two most common genes responsible for cystinuria, SLC7A9,^36^ and SLC3A1^36^ and two autosomal dominant genes SLC34A1^33^ and *SLC9A3R1*.^33^

Variants in APOL1 have been found to increase the risk of CKD in Black populations. Those carrying two APOL1 risk alleles have almost a 30-fold increased risk of CKD and cardiovascular-related mortality (odds ratio 1.8).^37–39^ Genovese, Friedman, and Pollak^39^ found variants on two specific loci in most population with recent African ancestry^34,39,40^—the G1 and G2 loci in APOL1. The G1 locus has with two coding variants—rs73885319 (p.S342G) and rs60910145 (p.I384M) in the last exon of APOL1 gene—while the G2 locus is a six base pair deletion close to G1, which removes amino acids N388 and Y389 in the APOL1 gene.^41,42^ These renal risk variants (RRVs) conferred enhanced innate immunity against African trypanosomes that causes the deadliest form of sleeping sickness. However, they simultaneously conferred the highest risk of hypertension-associated end-stage kidney disease (7–10-fold increase), focal segmental glomerulosclerosis (FSGS) (10–17-fold increase), HIV-associated nephropathy (29-fold increase), and other variants of nondiabetic kidney disease.^39,41,42^ The Kidney Allocation System^43^ was set up to improve renal allograft survival rates by improving the matching of recipient with donor kidneys. The Kidney Donor Risk Index (KDRI)^44^ is an integral aspect of the Kidney Allocation System to determine graft life span and estimate donor quality. It is a scoring system to quantify graft failure risk on the basis of a series of 10 deceased donor factors, one of which is a variable that asserts Black ethnicity/race. Although kidney transplantation seems to be the preferred treatment for patients with APOL1-associated nephropathy, it was found that Black donor kidneys were at a higher rate of failure after transplantation as compared with donors from other races.^45^ Retrospective studies^46^ acknowledge that the variation in donor APOL1 genotypes and not the Black ancestry is in fact responsible for the shorter allograft survival.

The APOLLO network protocol was created to help improve survival rates by improving policies for matching donors with recipients such that graft survival can be prolonged.^45^ Consequently, the role of genetic testing is increasing in renal transplantation to assess donor risk, recipient disease characterization, and customization of therapeutics using pharmacogenomic information. Marin and colleagues, in an extensive review,^47^ explored the increasing role of genetic testing in kidney transplantation. Because APOL1 RRVs are found in both donor and recipients, there is divided opinion on the extent of effect of the RRVs in donor vs recipients on the survival of graft recipients. Although Zhang and colleagues^48^ show that recipient APOL1 risk variants negatively affect kidney allograft survival and T-cell–mediated rejection rates, independent of donor APOL1 genotype or recipient ancestry, Freedman and colleagues document findings^46^ where RRVs from donors led to shorter renal allograft survival in a single-center study.

Mitochondrial dysfunction can also precede and participate in the pathophysiology of AKI through renal damage. In addition, mutations in the coenzyme Q10 biosynthesis pathway or mutations in the mtDNA 3243 A>G were found to be causal variants for tubular dysfunction and, subsequently, glomerular disease.^49^ It has become an open question whether this is addressable by the recently proposed mitochondrial transplantation^50^ in the tissue thought to promote regeneration and repair of the renal tissues.^45,51,52^

The past 5 years have seen activity in the advocacy and promotion of genetic testing for kidney diseases. Nestor and colleagues highlighted^53^ the rationale to introduce genetic testing in nephrology for adult and pediatric patients with CKD and the return of actionable results to patients as a first-in-line tool for diagnostics. In the same article, they also highlighted the need to understand the implications of receiving genetic test results for patient outcomes, examine the logistics of setting up routine genetic testing, and learn best practices to guide a clinician's decision making in the light of genetic test results. Around the same time, a group of physicians documented the effect of implementing a Renal Precision Program^54^ across a multihospital health system, wherein clinical care for CKD was guided by clinical genetic and pharmacogenomic predictors for CKD. They found that although clinicians agreed that genetic test results were indeed valuable in CKD risk stratification, they were uncomfortable discussing genetic results with patients possibly due to lack of exposure and education of providers in clinical genetics. Nestor and colleagues also developed a pilot study workflow^55^ that aims to return medically actionable findings from clinical genetic results to adult nephrology patients and identified key challenges in the workflow to return results to patients of which physician knowledge gap in genomics was recurrent. Establishing periodic educational conferences and standardized consultation notes along with connecting nephrologists with expert clinicians requiring extranephrologic referrals helped develop a more optimized workflow.

The use of clinical diagnostics, such as the reporting of causal variants, has increased the possibility of definitive diagnoses even in atypical cases of atypical disease around the world. The Australia and New Zealand Renal Gene Panels (ANZRGP) service^56^ at the Children's Hospital at Westmead used an accredited, targeted exome sequencing for multigene panels, associated with more than 20 kidney disease categories and over 200 different genes curated according to the ClinGen criteria. A recent instance of reporting of variants on the *PKD1* gene in autosomal dominant polycystic kidney disease (ADPKD) turned out to be the first of its kind performed in a clinical setting using whole-genome sequencing.^57^ Jayasinghe and colleagues performed a clinically accredited singleton exome sequencing^58^ in 204 patients suspected with monogenic kidney disease to identify a molecular diagnosis in 40% of their patients, signifying substantial clinical utility. However, with the diversity in the number of sequencing technologies, gaining an understanding of the various genetic testing modalities is important to enable clinical nephrologists to maximize diagnostic yield while minimizing overhead costs. The ANZRGP panels had diagnostic yields^56^ as low as 13% for the congenital anomalies of the kidney and urinary tract (CAKUT) panel for pathogenic or likely pathogenic variants. Another large clinical cohort analysis to assess the diagnostic efficacy of exome sequencing in patients with CKD^59^ shed light on the surprisingly low diagnostic yield, at around 10% for almost 3000 patients. Although this yield is lower than the diagnostic yield as documented by Jayasinghe *et al.*,^58^ it highlights the dependence of yield on patient selection and the level of genetic-phenotypic characterization of the disease in question.

Aiming to understand how to integrate a successful genomic diagnostics element into an existing nephrology practice, Cocchi *et al.* elaborated on the factors^60^ that need to be considered to implement this powerful diagnostic tool with reduced overhead costs. Significant factors include choosing the most appropriate sequencing modality and encouraging and establishing multidisciplinary partnerships across multiple departments to manage diagnosis and patient care. Lundquist and colleagues documented their successful efforts^61^ into establishing and incorporating a *de novo* genetics clinic into an ambulatory nephrology care with minimal capital investment. They stated that a focused exome panel along with a novel service delivery model that optimized limited Clinical Genetic Counseling (CGCs) resources when needed helped make their first year a successful venture and limited out-of-pocket costs to patients. Thomas and colleagues identified a workable solution^62^ by means of establishing a multidisciplinary Renal Genetics Clinic and partnering with multidisciplinary teams including CGCs to enhance the workflow of routine genetic testing for patients with kidney disease. Inclusion of CGCs, partnerships with clinicians and genomic experts outside of nephrology to provide support in the light of a diagnosis along with education of patients and families about the significance of the genetic test and its outcomes helped improve diagnostic rates and elevate patient care and disease management.^62–66^

The comparative increase in insufficient or contradictory evidence has limited the efficacy of genomic diagnostics in a clinical context.^28^ This has been either due to lack of relevance in diagnostic accuracy or due to misclassification of variants when associating rare variants (stemming from small sample sizes). Specifically, the availability of accurate data to correctly estimate prior probability to establish the prevalence of detectable disease in a population is necessary to prevent potential misinterpretation of genetic data^28^ and misclassification of genetic variants. For this purpose, the Clinical Genome Resource (ClinGen) with kidney disease–related subgroups (ClinGen Kidney Disease Working Group or CDWG) was founded to provide a clinical resource and define protocols and rules to identify clinically relevant genes and variants.^67^ The CDWG would include a series of expert panels, such as the Kidney Cystic and Ciliopathy Disorders Gene Curation Expert Panel,^68^ in various disease groups including the development of expert panels to tackle various kidney glomerulopathies, ciliopathies, and tubulopathy.

Of course, nongenetic factors (and the data that capture them) that also affect the outcome for kidney disease are crucial in diagnosis, treatment, and management of kidney disease. The environment itself can be a risk factor,^69^ while toxin-induced nephropathies such as those from cadmium and aristolochic acids can cause kidney injury with proteinuria and chronic, progressive tubulointerstitial kidney disease, respectively.^69^ The relative contribution of genetics and environment to a given disease or disease class arises naturally in precision medicine, and the question of “genetic code *versus* zip code” is an open one in precision nephrology. Some quantification is possible. For instance, in high-resource settings—such as large urban hospitals with highly trained medical personnel, advanced monitoring capabilities, and academic research centers^70^—the etiologic spectrum of patients developing AKI in such scenarios is similar to hospital-acquired AKI (HAAKI) and is comparatively more “preventable and treatable” primarily due to continuous monitoring and advanced management of this condition.^70–72^ In comparison, in low-resource settings such as health care centers with limited financial and academic resources, more patients progress to more severe forms of AKI.^71,72^ This could happen due to the lack of timely medical interventions and unavailability of advanced diagnostic methods or even monitoring or dialysis. In such scenarios, AKI is considered to be primarily community-acquired AKI, which can also be acquired from infections, nephrotoxic medications, and other environmental exposures.^70,71^

The fusion of omics data and clinical data has enabled novel insights derived from precision medicine and public health techniques.^15^ The use of publicly shared health information through social media networks helps cluster similar patient profiles across state and international borders to enrich knowledge and information about poorly defined patient conditions and phenotypes.^73,74^ Patients increasingly share their health and clinical experience online and reach out to support groups or networks of families with patients.^75^ This is specifically true in the case of patient families with a rare disease diagnosis or a disease, which may yet be undiagnosed.^73,76^ Another transformative shift in the study of predictive factors for diseases is the use of environmental data, such as air and water quality, pollution, crime rates, and temperature changes.^15,77^ Epigenetic factors add selective weight to existing diagnostic rules because of the effect they have on some populations for making them more vulnerable to specific disease conditions.

## Precision Nephrology in Clinical Therapeutics

The generation of Big Data in genomics and population health is being adapted into personalized diagnostics and therapeutics especially in the areas of kidney disease diagnosis, treatment, and management. For example, previous studies of diabetes showed improved outcomes using SGLT2 inhibitors.^78,79^ By broadening the focus of this study from outcomes to other aspects of general health, they found effects on kidney health as well. This next section will help us illuminate our second aim: *optimizing general health with respect to the kidney in a data-driven manner.*

The kidney plays an important role in the metabolism of nutrients for overall health. In patients with CKD, this ability to maintain homeostasis and excrete harmful solutes out of the body is restricted, leading to worsening kidney and overall health. Thus, modification of nutritional intake^80^ becomes crucial to improve general health and reduce the poor outcomes for the kidneys.^81^ The generation of new data is enabling a more tailored, precise approach to these modifications: For instance, the Chronic Renal Insufficiency Cohort (CRIC) longitudinal study^82^ is studying time course and protein energy status of patients with CKD progressing toward ESKD.

Clinical trials are highlighting novel interventions as well: In the CREDENCE study, a recent phase 3 clinical trial, the use of SGLT2 inhibitors for type 2 diabetes patients with CKD helped reduce the risk of renal replacement therapy. Consequently, death caused by kidney insufficiency fell by almost 33%, despite doubling serum creatinine.^10^ In addition, in another major multicenter placebo-controlled, three-year trial, TEMPO, a vasopressin-2 receptor antagonist was shown to slow the increase in total kidney volume and the decline in kidney function and slow cyst growth.^83^ This drug was developed by using genomic information from patients with ADPKD.^84^ However, because 15% of the participants in the drug group discontinued the drug due to liver function abnormalities,^83^ it is clear that pharmacogenetics will be critical in successful adoption of precision therapeutics in nephrology. Another trial, REPRISE, tested the safety and efficacy of the drug; it was shown to help reduce estimated glomerular filtration rate (eGFR) decline by almost 35% following which the FDA approved the drug in adult patients for rapidly progressing ADPKD.^84,85^

IgA nephropathy (IgAN) (also known as Berger disease) is the most common form of glomerular nephropathy, and prognosis is poor.^86,87^ It occurs when immunoglobulin A (IgA) builds up in the kidneys, progressing to ESKD in almost 50% of the affected cases. The International IgA Nephropathy Network was created by group of clinicians from the Renal Pathology Society interested in IgAN back in the early 2000s.^88^ They established a working group of nephrologists and scientists from across the world to develop an internationally accepted consensus classification of IgAN.^89^ The outcome was a histopathologic scoring system known as the MEST score^90^ on the basis of specific clinicopathologic features that could independently predict the outcome in IgAN. In 2016, the IgAN Classification Working Group updated the score to include crescents, which are also predictive of outcome to create the MEST-C score. On the basis of this score, they developed a large knowledgebase of clinical datasets from well-characterized adults and children with IgAN.^91^ In 2019, Barbour and colleagues from the International IgA Nephropathy Network derived and externally validated a clinical risk prediction model using patient clinical characteristics including the MEST-C histologic scores, age, and medication use with or without race/ethnicity information.^92^ The models with the MEST-C scores were shown to be accurate in the prediction of disease progression and risk stratification in IgAN along with positive implications toward clinical trials and biomarker research. This prediction tool was further updated with a multiethnic cohort and externally validated to be able to predict patient risk stratification two years postbiopsy.^93^ In addition to the IgAN prediction tool, other tools such as a long-term survival model developed by Li and colleagues^94^ to gain an insight into the increased risk induced by specific patient characteristics highlight the breakthroughs in the understanding of renal progression and subsequent therapies for treatment. Identification and validation of genomic markers is underway to create screening panels for individuals at higher risk of IgA as well as the development of tools for prediction of disease prognosis and methods for earlier intervention to prevent the recurrence of IgAN.^95^

For genetic risk, the genotype-phenotype associations for the gene apolipoprotein L1 (APOL1) have been a pivotal moment for precision nephrology. Despite significant risk, further research is needed to understand why not all APOL1 variant carriers contract kidney disease. APOL1 *has* been genetically validated as a target gene for kidney disease, and thus, pharmacologically regulating the expression of *APOL1* at an individual level is an important problem. Clinical trials are currently underway to attempt to capitalize on this variability into personalized, targeted therapies for FSGS and management of kidney disease.^45,96–98^

**Figure 1:**
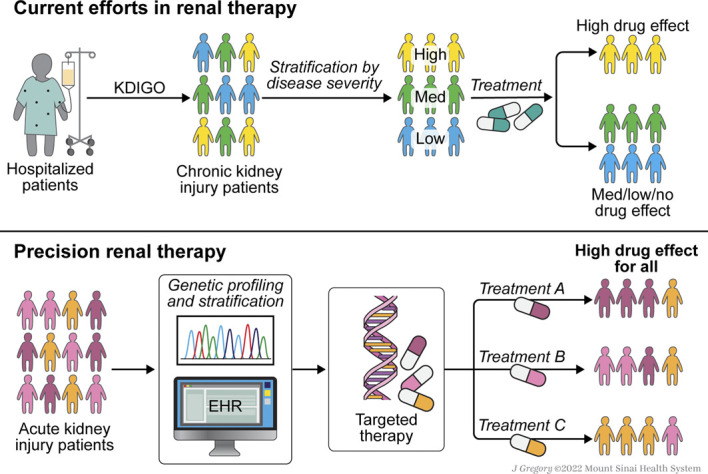
**Stratification by severity of disease results in suboptimal treatment outcomes.** Involvement of precision renal therapy allows the stratification to also include genetic information from large-scale population data to develop targeted therapy. This approach promises an improved set of treatment outcomes as compared with prior approaches.

Omics research, and clinical trials that include genomic data, have the potential to inform more personalized nephrology care and improve patient outcomes. However, as highlighted by some of these trials, such precision medicine is at best partially effective when only considering kidney-specific risk factors. This is the case in part because kidney dysfunction may arise as a result of comorbidities or dysregulation or injury in other organs.^3,99^ This complex interdependence thus makes it necessary to focus on optimization of overall health to minimize dysregulation in kidney function.^100–102^ Inclusion of other factors that affect an individual's overall health is an important aspect of precision medicine and precision nephrology (Figure 1).

## Impact of Race in Precision Nephrology

The implementation of personalized medicine in clinical decisions needs to consider a wide spectrum of heterogeneous data, including family history, nutrition, lifestyle, environmental effects, observed phenotypic traits, genetic/genomic results, and clinical measurements. For example, Adigbli, in a review about the inclusion of race in precision medicine,^103^ observed that commonly observed stressors that stem from systemic racism tend to confound these major determinants of health and concentrate the burden of disease complexity in minority populations. He continued to state^103^ that the complications from the disease and disease management have a heavier effect on social and economic statuses for such populations.

The skewed affliction toward these populations tends to incorrectly reflect this as a true genetic difference. Hsu *et al.* tried to determine whether they could calculate the eGFR—a value that has historically been used to determine the risk of CKD in patients—by using race as a covariate in the equation.^104^ They found that there was a correlation between African ancestry and the serum creatinine levels. They suggested using genetic ancestry information rather than race-based categorization that does not reflect ancestry to avoid exacerbating the systemic discrimination. In essence, using self-reported race in predictions for precision nephrology blurs the precision of such predictions. Race alone misses the recognition of substantial diversity arising from the inherent genetic variability within self-reported ethnic subpopulations.

The National Kidney Foundation (NKF) and American Society of Nephrology (ASN) have created a joint task force^105^ to examine the implications of race in the diagnosis and management of patients with or at risk for kidney disease. Removal of self-reported race from the models that calculate the key risk stratification criteria was believed to enable an unbiased assessment of kidney function and more personalized care for patients with kidney disease. However, Obermeyer *et al.* in a recent article^106^ showed that a widely used commercial algorithm was recommending fewer Black patients (who were evidently sicker) for additional care than White patients at the same risk category. The algorithm was erroneously predicting need for additional care on the basis of health care expenditure costs without accounting for disparities among access to health care, a fact which is rooted in systemic bias. They observed that the removal of reported race from their model does not result in an unbiased prediction algorithm because other variables that reflected racial bias such as patient health care costs that seemed to be a proxy for health had crept into the model. They conclude that a significant change in the kind of labels the model is fed needs to be seriously reconsidered to prevent a pernicious reintroduction of bias. Some of these critical issues go back to how algorithms are designed. Murray *et al.* talk about their experience^107^ with a popular AI patient scheduling assist tool that would incorrectly predict which patients could most likely be a “no show” because of explicit and implicitly biased feature variables in the model. Features such as prior no shows (most likely related with low socioeconomic status), work or child care commitments, and chronic health issues could exacerbate the likelihood of lateness and have a higher likelihood of being tagged as a “no show” just to have their appointment canceled leaving the already sick patient in jeopardy. Instead, they decided to build their own tool with patient-positive interventions, such as increasing flexible scheduling times and assistance with transportation and childcare, which when deployed, reduced patient no show rates by 9 percent. Ghassemi *et al*.^108^ correctly point out that humans are imperfect, and bias is a human imperfection also defined as systemic neglect. Bias always creeps into the systems we build, and therefore, these systems cannot be perceived to be equitable. Clinicians and scientists who develop such systems will need to recognize historic and racial injustices against marginalized groups and will need to work to implement policies and practices that identify the implicit biases that also creep into measurement systems. Ultimately, a precisely labeled data-driven approach along with policies to ensure the identification and mitigation of bias in measurement, training, and testing of such models will enhance the power of algorithmic predictions to uncover valuable patterns that benefit public health.

## The Integration of Multimodal Big Data with Machine Learning in the Clinic

To many, *precision medicine* connotes genomics and genetic sequencing. However, although genomics is a critical component of precision medicine, the optimization process in precision medicine benefits from more resolution than genetic data alone can provide. This may include combining data from multiple modalities: data from laboratory results, clinical electronic health record (EHR) data, transcriptomic data, metabolomic data, metagenomic data, and the wealth of data available in biomedical research. An important step toward this, The National Institute of Diabetes and Digestive and Kidney Diseases (NIDDK) Kidney Precision Medicine Project (KPMP), focuses on obtaining and evaluating human kidney biopsies from patients who suffer from AKI or CKD to create a comprehensive Kidney Tissue Atlas, defines disease subgroups, and identifies critical cells, pathways, and targets for novel therapies.^109,110^ KPMP has also helped develop two specific ontologies, the Kidney Tissue Atlas Ontology (KTAO) and the Ontology of Precision Medicine and Investigation (OPMI). These ontologies aid with the creation of the tissue atlas to specifically annotate kidney data. These ontologies also continue to revise existing definitions for kidney disease within precision medicine. By enriching molecular, transcriptomic, and metabolomic features with granular clinical data, KPMP hopes to uncover new subtypes and to redefine classification and categorization for kidney disease. The goal is for these subtypes to elicit better outcomes with novel therapeutic approaches.

**Figure 2:**
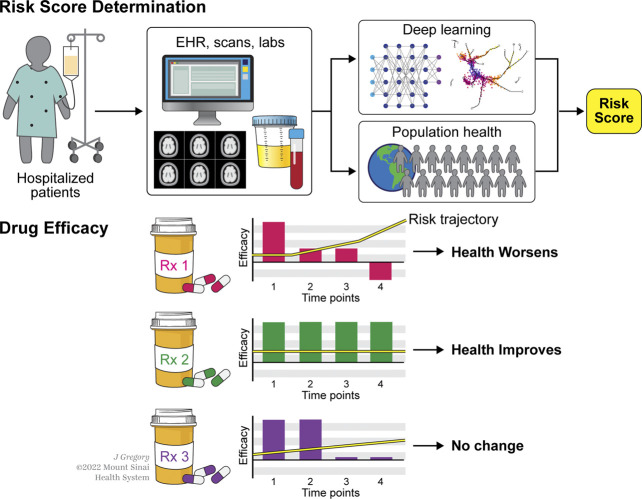
Implementation of a multimodal data-driven approach shows a more optimal strategy in not just diagnosis of current condition and treatment but a longitudinal effort in understanding the risk of future disease progression as well.

**Figure 3:**
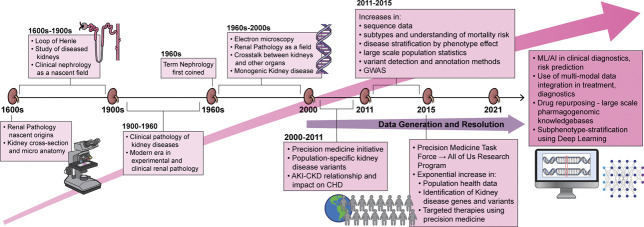
**Tracing the arc of precision nephrology that bends toward increasing data at increased resolution fueling innovation**.

The availability of multiple high-resolution data modalities opens avenues for fast, accurate diagnosis and early prediction of risk for kidney disease complexities. For instance, high-resolution population health may be leveraged for longitudinal modeling and prediction of patient health. Machine learning (ML) algorithms are now being used in this context for both acute and CKD. The electronic health record can be combined with the patient's genomic, transcriptomic, metabolomic, physiological, and imaging to generate a precise score unique to the individual. In a multicenter retrospective cohort study, a team at the National Clinical Research Center of Kidney Diseases, China, developed a risk prediction algorithm using a supervised machine learning model known as eXtreme Gradient Boosting (XGBoost) for risk and long-term outcomes for IgAN.^86^ The artificial intelligence (AI)-based startup Renalytix uses a platform KidneyIntelX to integrate multimodal data for early prognostication.^111^ In an Artificial Intelligence in Renal Scarring (AIRS) study, machine learning models for noninvasive quantification of kidney fibrosis from imaging scans have been developed^112^ (Figure 2). The Singapore Epidemiology of Eye Diseases used a multiethnic cohort to develop and validate a deep learning algorithm^113^ that can detect CKD using retinal images. Google's DeepMind^114^ developed a deep learning framework, a recurrent neural network (RNN) operating sequentially over thousands of electronic health records, to build an internal memory that keeps track of relevant information seen up to that point. At each time point, the model would then output a probability of AKI occurring at any stage of severity within the next 48 hours. The model combined patient EHR data with creatinine levels from blood, potassium, age, heart rate, and oxygen saturation to predict an early onset of AKI at least 48 hours before a physician could detect it. This early warning gives physicians more time to intervene and manage disease severity efficiently. The advancement in wearable technology adds real-world longitudinal data from personal smart devices (Figure 3).

Data generated from these devices are only currently being explored by large research groups trying to incorporate this information with population health to predict health trajectories. A recent study used Apple Watch's data tracking to monitor and warn of the risk of incidence of COVID-19.^115^ Another feasibility study validated the use of wearable trackers to warn early of clinical deterioration in patients.^116^ These initial studies portend the potential that is present in multimodal data. This shift in use of longitudinal big data shows promise in future disease monitoring and risk prediction.

## The Arc of Precision Medicine in Kidney Disease is Long but Bends toward Innovation

The next decade in Precision Nephrology can be envisioned as a case where AI/ML are deeply integrated in clinical practice and will include prediction, prognosis of significant disease risks, and long-term estimates of living with the disease. The ability to incorporate increasingly noninvasive modes of measurement of data such as urine, saliva, sweat, and continuously track disease risk by means of wearable and other data will be valuable for improving patient care. The use of AI to collect, clean, normalize, and track electronic medical, dental, and imaging records for the patient population is an obvious opportunity that is currently being developed across the world.^111,117,118^

There is a need for more initiatives that train nephrologists to develop frameworks to incorporate AI and ML in their routine diagnostics.^119,120^ With increasing aggregation of complex multimodal data, the challenges in implementing supervised ML range from bias in performance evaluation to skewed predictions due to overfitting or underfitting of the model. It is essential to learn how to identify and implement only those models that have clear performance metrics, with little to no bias, and show rigor in testing and validation for clinical purposes. In fact, there is now a better understanding of the common pitfalls that occur when implementing supervised machine learning in clinical practice.^121^ An article published in *JAMA* recently reviewed more than 70 publications that compared diagnostic accuracy of ML models implemented in the clinic against equivalent decisions made by doctors and uncovered a significant bias in the training area originating only from three states in the country.^122^ The increasing numbers of scientists and physicians working with AI/ML algorithms have emphasized the need to detect, correct, and mitigate biases at all levels. One possible approach could be the establishment of ethics teams within institutions to help with identification and limit such biases from creeping into these systems.

The increasing roles of ML and AI in health care diagnostics and prognosis should be used to *augment* (rather than automate) critical decisions in the clinic, and they should be driven by unbiased, representative datasets. The foray of deep learning into clinical care shows promise in leveraging AI to extract heterogeneous, temporal information from EHR data for the development of risk stratification, predictive analytics, and clinical decision support, and it has ushered in a new era in the development of Intelligent Learning Health Systems.^123^ These systems will be self-sustaining only to the extent that they can successfully convert data to knowledge and knowledge to wisdom.

While “AI in precision nephrology” turns from idea to reality, it is important to remember that the success of improving patient outcomes has always relied on a fusion of scientific rigor, human compassion, daring imagination, critical thinking, thoughtful leadership, multidisciplinary teamwork, and a *healthy dose of luck*!
